# Data set of phylogenetic analysis inferred based on the complete genomes of the family *Nodaviridae*

**DOI:** 10.1016/j.dib.2016.08.025

**Published:** 2016-08-20

**Authors:** Chen-Fei Low, Hamidun Bunawan

**Affiliations:** Institute of Systems Biology, Universiti Kebangsaan Malaysia, 43600 UKM Bangi, Selangor, Malaysia

**Keywords:** Phylogenetic, Nodaviridae, Multiple sequence alignment, Complete genome

## Abstract

In this article, nine complete genomes of viruses from the genus *Alphanodavirus* and *Betanodavirus* (Family *Nodaviridae*) were comparatively analyzed and the data of their evolutionary origins and relatedness are reported. The nucleotide sequence alignment of the complete genomes from all species and their deduced evolutionary relationships are presented. High sequence similarity within the genus *Betanodavirus* compared to the genus *Alphanodavirus* was revealed in multiple sequence alignment of the *Nodaviridae* genomes. The amino acid sequence similarity for both RNA1 and RNA2 ORF is more conserved in *Betanodavirus*, compared to *Alphanodavirus*. The conserved and variable regions within the virus genome that were defined based on the multiple sequence alignments are presented in this dataset.

**Specifications Table**

**Table:** 

Subject area	Biology
More specific subject area	Virology; Molecular evolution
Type of data	Figures, tables
How data was acquired	Complete genome sequences were retrieved from a public database (*NCBI*)
Data format	Analyzed
Experimental factors	Complete genome sequences were retrieved from *NCBI*
Experimental features	Multiple sequence alignment and construction of phylogenetic trees using *MEGA7.*
Data source location	N/A
Data accessibility	Data is within this article

**Value of the data**

• Multiple sequence alignment established the conserved and variable regions within the *Nodaviridae* genomes are essential to study the virus evolution and the virus diversity.
• The conserved and variable regions in the *Nodaviridae* complete genome defined by multiple sequence alignment allowed the design of sequence specific primers that can be used for virus detection and identification.
• Virus identification and classification inferred based on phylogenetic analysis of sequence conservation in *Nodaviridae* is applicable in subsequent downstream research.

## 1 Data

**Fig. 1:**
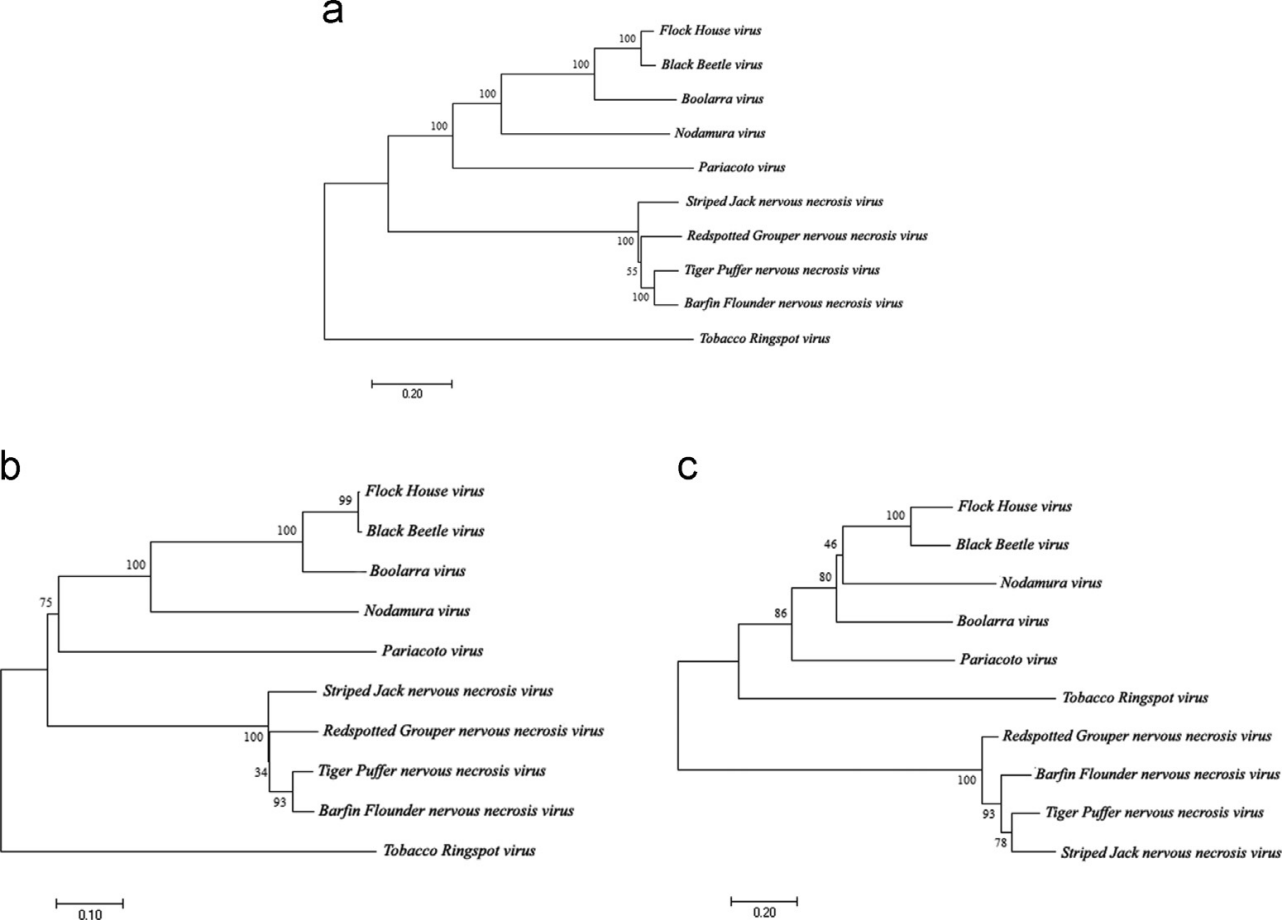
The evolutionary history of (a) *Nodaviridae* genomes; (b) sub-genome RNA1; and (c) sub-genome RNA2 were inferred using the Neighbor-Joining method. Percentage of replicate trees of 1000 replicates bootstrap test is presented next to the branches.

**Fig. 2:**
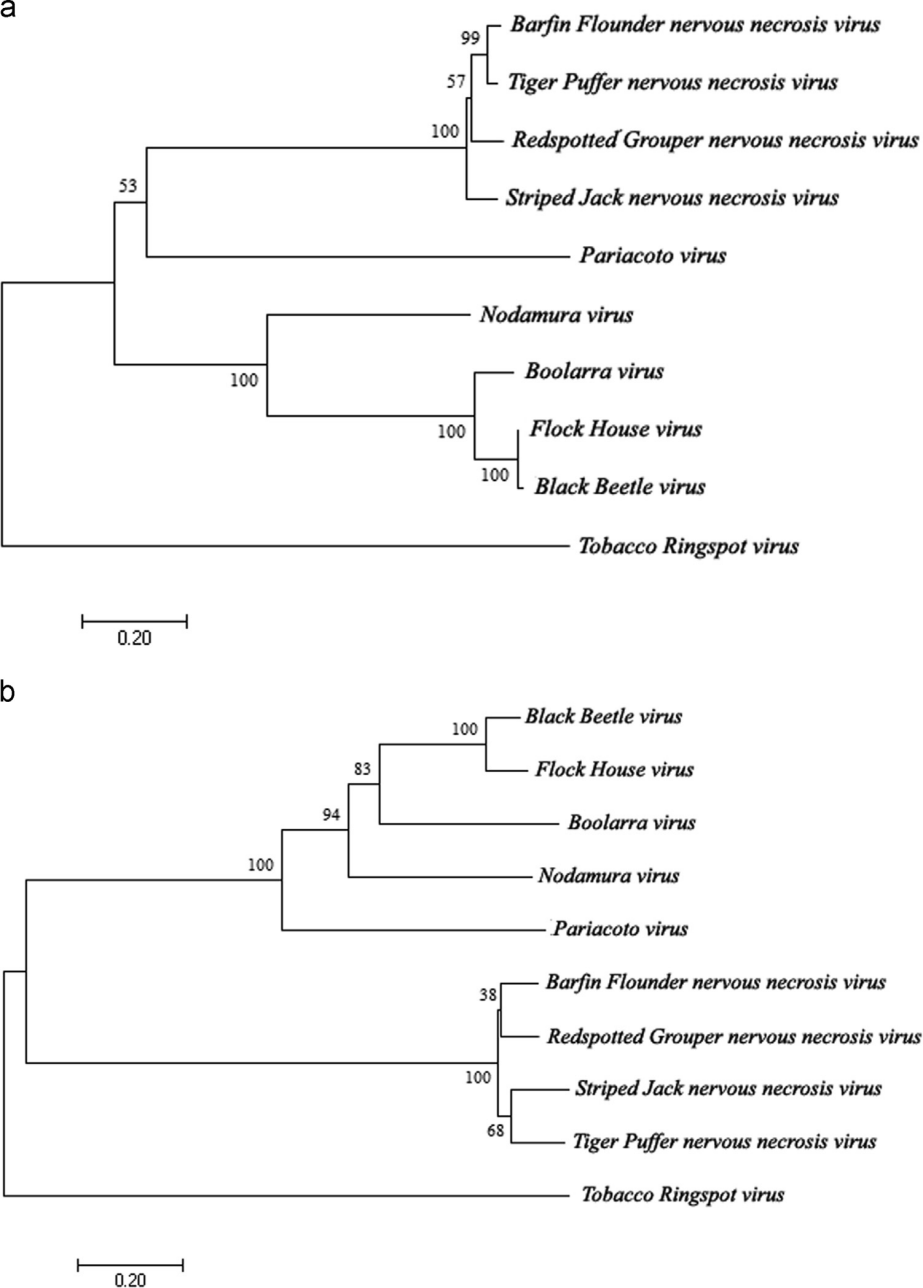
The evolutionary history of deduced amino acid sequence of (a) sub-genome RNA1 ORF; and (b) sub-genome RNA2 ORF were inferred using the Maximum-Likelihood method. Percentage of replicate trees of 1000 replicates bootstrap test is presented next to the branches.

Complete genomes of nine *Nodaviridae* species available in *NCBI* database were aligned using *MEGA7* software [1]. Based on the alignment, phylogenetic trees [2] were constructed (Fig. 1, Fig. 2). Data on sequence similarity percentage [3], [4] are presented in Table 1, Table 2, Table 3.

## 2 Experimental design, materials and methods

The complete genome data of *Nodaviridae* were retrieved from *NCBI* (http://www.ncbi.nlm.nih.gov/). Nine *Nodaviridae* species in the family of *alphanodavirus* and *betanodavirus* were selected (Table 1) for alignment. *Tobacco ringspot virus* was included as outgroup for phylogenetic analysis. The open reading frames (ORFs) of genomic RNA1 and RNA2 were identified using ORF Finder (http://www.ncbi.nlm.nih.gov/orffinder/) and the deduced amino acid sequences were aligned using *MEGA7*. Phylogenetic trees based on virus genomes and deduced amino acid sequences of ORF were constructed by Neighbor-Joining and Maximum Likelihood respectively (Table 4).

## Figures and Tables

**Table 1:** Accession numbers for the sequences used in this study.

*Nodaviridae* species	Accession numbers
*Black beetle virus*	RNA1: NC_001411.2
RNA2: NC_002037.1
	
*Flock house virus*	RNA1: NC_004146.1
RNA2: NC_004144.1
	
*Nodamura virus*	RNA1: NC_002690.1
RNA2: NC_002691.1
	
*Boolarra virus*	RNA1: NC_004142.1
RNA2: NC_004145.1
	
*Pariacoto virus*	RNA1: NC_003691.1
RNA2:NC_003692.1
	
*Striped Jack nervous necrosis virus*	RNA1: NC_003448.1
RNA2: NC_003449.1
	
*Tiger Puffer nervous necrosis virus*	RNA1: NC_013460.1
RNA2: NC_013461.1
	
*Barfin Flounder nervous necrosis virus*	RNA1: NC_013458.1
RNA2: NC_013459.1
	
*Redspotted Grouper nervous necrosis virus*	RNA1: NC_008040.1
RNA2: NC_008041.1
	
*Tobacco Ringspot virus*	RNA1: NC_005097.1
RNA2: NC_005096.1

**Table 2:** Percent identity matrix of deduced amino acid sequence of sub-genome RNA1 and RNA2 ORF.

Deduced amino acid sequence of sub-genome RNA2 ORF	Deduced amino acid sequence of sub-genome RNA1 ORF									
		*Alphanodavirus*					*Betanodavirus*			
		*Black beetle virus*	*Flock house virus*	*Nodamura virus*	*Boolarra virus*	*Pariacoto virus*	*Striped Jack nervous necrosis virus*	*Tiger puffer nervous necrosis virus*	*Barfin flounder nervous necrosis virus*	*Redspotted grouper nervous necrosis virus*

	*Black beetle virus*	…	99.00	42.16	83.87	21.18	22.75	22.75	22.86	23.09
	*Flock house virus*	86.73	…	42.57	84.27	21.29	22.86	22.86	22.97	23.20
	*Nodamura virus*	48.72	47.70	…	40.24	24.15	25.38	26.36	25.84	26.14
	*Boolarra virus*	52.55	51.02	45.50	…	22.42	22.75	23.09	23.20	23.64
	*Pariacoto virus*	39.33	38.82	38.32	35.29	…	29.03	28.51	28.21	27.95
	*Striped Jack nervous necrosis virus*	19.15	18.79	16.31	18.25	16.79	…	87.78	87.97	87.88
	*Tiger puffer nervous necrosis virus*	18.44	17.38	17.02	18.60	17.52	80.88	…	95.41	88.70
	*Barfin flounder nervous necrosis virus*	22.50	21.07	18.21	20.14	19.49	81.07	81.66	…	88.48
	*Redspotted grouper nervous necrosis virus*	20.36	18.93	17.50	20.14	18.01	81.66	81.66	86.69	…

**Table 3:** Percent identity matrix of nucleotide sequence of sub-genome RNA1 and RNA2.

Nucleotide sequence of sub-genome RNA2		Nucleotide sequence of sub-genome RNA1								
		*Alphanodavirus*					*Betanodavirus*			
		*Black beetle virus*	*Flock house virus*	*Nodamura virus*	*Boolarra virus*	*Pariacoto virus*	*Striped Jack nervous necrosis virus*	*Tiger puffer nervous necrosis virus*	*Barfin flounder nervous necrosis virus*	*Redspotted grouper nervous necrosis virus*

	*Black beetle virus*	…	99.00	48.88	78.12	37.29	39.26	38.95	38.88	40.27
	*Flock house virus*	79.17	…	49.04	78.15	37.50	39.37	39.15	39.05	40.44
	*Nodamura virus*	51.79	50.30	…	47.87	39.36	39.34	39.98	39.63	39.23
	*Boolarra virus*	54.25	53.96	49.59	…	37.59	39.18	38.54	38.03	39.40
	*Pariacoto virus*	46.76	44.83	46.79	43.58	…	44.19	44.56	43.49	43.28
	*Striped Jack nervous necrosis virus*	37.34	36.81	35.19	33.33	34.98	…	82.14	82.67	81.75
	*Tiger puffer nervous necrosis virus*	37.42	39.10	36.26	34.88	34.73	81.77	…	91.26	82.22
	*Barfin flounder nervous necrosis virus*	38.17	37.87	35.58	35.28	35.79	78.38	79.73	…	82.68
	*Redspotted grouper nervous necrosis virus*	38.03	38.59	35.69	34.29	36.64	79.12	79.76	82.11	…

**Table 4:** Percent identity matrix of *Nodaviridae* complete genome.

	Complete genome of *Nodaviridae*								
	*Alphanodavirus*					*Betanodavirus*			
	*Black beetle virus*	*Flock house virus*	*Nodamura virus*	*Boolarra virus*	*Pariacoto virus*	*Striped Jack nervous necrosis virus*	*Tiger puffer nervous necrosis virus*	*Barfin flounder nervous necrosis virus*	*Redspotted grouper nervous necrosis virus*

*Black beetle virus*	…	92.85	49.73	71.35	39.59	37.25	37.72	37.89	37.69
*Flock house virus*		…	49.49	71.31	39.81	36.65	37.82	37.74	37.43
*Nodamura virus*			…	48.50	39.98	36.81	38.11	37.98	37.25
*Boolarra virus*				…	38.46	37.20	37.25	37.63	36.68
*Pariacoto virus*					…	44.21	44.23	43.71	42.81
*Striped Jack nervous necrosis virus*						…	81.76	81.53	80.91
*Tiger puffer nervous necrosis virus*							…	87.94	81.67
*Barfin flounder nervous necrosis virus*								…	82.43
*Redspotted grouper nervous necrosis virus*									…

## Appendix A Supplementary material

Supplementary material: **SF1:** Multiple sequence alignment of *Nodaviridae* complete genome using ClustalW of *MEGA7.0.18*. Virus species are indicated at the left of sequence. Color gradients correspond to the percentage of conversed nucleotide residues.

.

Supplementary material: **SF2:** Multiple sequence alignment of *Nodaviridae* complete sub-genome RNA1 using ClustalW of *MEGA7.0.18*. Virus species are indicated at the left of sequence. Color gradients correspond to the percentage of conversed nucleotide residues.

.

Supplementary material: **SF3:** Multiple sequence alignment of *Nodaviridae* complete sub-genome RNA2 using ClustalW of *MEGA7.0.18*. Virus species are indicated at the left of sequence. Color gradients correspond to the percentage of conversed nucleotide residues.

.

Supplementary material: **SF4:** Deduced amino acid sequence alignment of *Nodaviridae* sub-genome RNA1 ORF using ClustalW of *MEGA7.0.18*. Virus species are indicated at the left of sequence. Color gradients correspond to the percentage of conversed amino acid residues.

.

Supplementary material: **SF5:** Deduced amino acid sequence alignment of *Nodaviridae* sub-genome RNA2 ORF using ClustalW of *MEGA7.0.18*. Virus species are indicated at the left of sequence. Color gradients correspond to the percentage of conversed amino acid residues.
